# Supplementary material for: HPF1-dependent PARP activation promotes LIG3-XRCC1-mediated backup pathway of Okazaki fragment ligation
Source: Nucleic Acids Res. 2021 Apr 19;49(9):5003–16. doi: 10.1093/nar/gkab269 (PMC8136790; doi:10.1093/nar/gkab269)
Supplement: gkab269_Supplemental_File [file gkab269_supplemental_file.pdf]

SUPPLEMENTARY DATA

HPF1-dependent PARP activation promotes LIG3-XRCC1-mediated backup pathway of Okazaki fragment ligation

Soichiro Kumamoto[1], Atsuya Nishiyama[1*], Yoshie Chiba[1], Ryota Miyashita[1], Chieko Konishi[1], Yoshiaki Azuma[2], and Makoto Nakanishi[1*]

[1] Division of Cancer Cell Biology, The Institute of Medical Science, The University of Tokyo, 4-6-1 Shirokanedai, Minato-ku, Tokyo 108-8639, Japan
[2] Department of Molecular Biosciences, University of Kansas, Lawrence, Kansas, 66045, U.S.A.

* To whom correspondence should be addressed. Tel: 81-3-5449-5341; Fax: 81-3-5449-5342; Email: mkt-naka@g.ecc.u-tokyo.ac.jp

Correspondence may also be addressed to Atsuya Nishiyama. Tel: 81-3-5449-5731; Fax: 81-3-5449-5342; Email: uanishiyama@g.ecc.u-tokyo.ac.jp

Figure S1

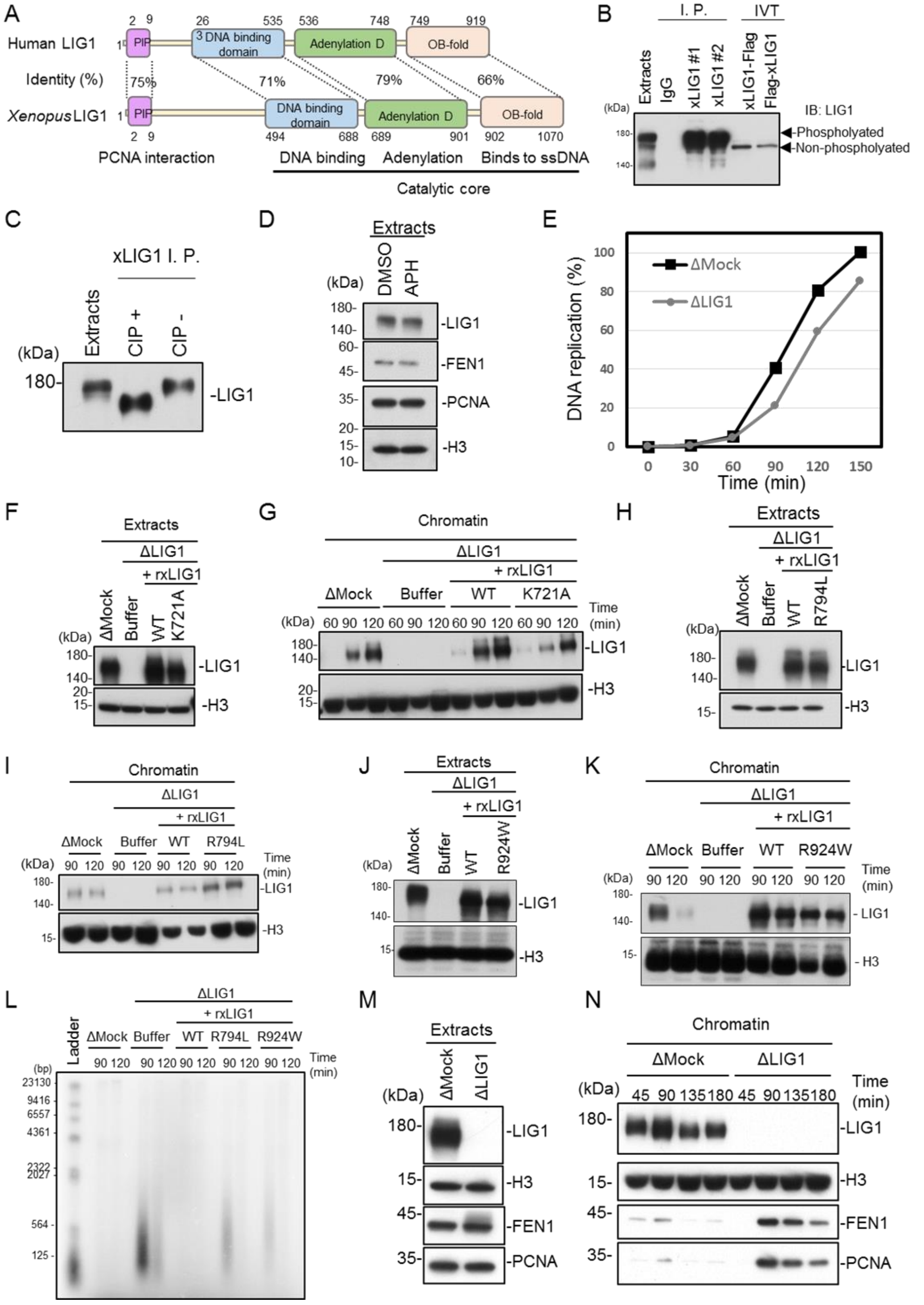
A
Human LIG1
Identity (%)
Xenopus LIG1
PIP
DNA binding domain
Adenylation D
OB-fold
75%
71%
79%
66%
PCNA interaction
DNA binding
Adenylation
Binds to ssDNA
Catalytic core
B
I. P.
IVT
Extracts
IgG
xLIG1 #1
xLIG1 #2
xLIG1-Flag
Flag-xLIG1
IB: LIG1
-Phospholyated
-Non-phospholyated
C
xLIG1 I. P.
Extracts
CIP +
CIP -
-LIG1
D
Extracts
DMSO
APH
-LIG1
-FEN1
-PCNA
-H3
E
ΔMock
ΔLIG1
DNA replication (%)
Time (min)
F
Extracts
ΔLIG1
+ rxLIG1
ΔMock
Buffer
WT
K721A
G
Chromatin
Time (min)
H
R794L
I
J
R924W
K
L
Ladder
M
N

Supplementary Figure 1. (A) Domain structures of *Xenopus* and Human LIG1. LIG1 is composed of a PCNA-interacting protein (PIP) motif, a DNA binding domain, an adenylation domain and an oligonucleotide/oligosaccharide-binding (OB)-fold domain that are well conserved in humans and *Xenopus*. (B) Immunoblot analysis of egg extracts, LIG1 immunoprecipitates and reticulocyte lysates translating recombinant FLAG-tagged xLIG1 (n = 2). (C) xLIG1 protein was immunoprecipitated with the anti-xLIG1 antibody and incubated with (+) or without (–) alkaline phosphatase. (D) The extracts used in the chromatin binding experiment shown in Figure 1D were analyzed by immunoblot using the indicated antibodies. (E) DNA replication in xLIG1- and mock-depleted extracts. The relative amounts of DNA synthesis are shown (n = 2). (F) LIG1-depleted extracts were supplemented with wild-type xLIG1 or xLIG1-K721A and analyzed by immunoblotting using the indicated antibodies. (G) The extracts from (F) were used to replicate sperm nuclei. Chromatin-bound proteins were analyzed by immunoblotting (n = 3). (H) LIG1-depleted extracts were supplemented with wild-type xLIG1 or xLIG1-R794L and analyzed by immunoblotting using the indicated antibodies. (I) The extracts from (H) were used to replicate sperm nuclei. Chromatin-bound proteins were analyzed by immunoblotting (n = 2). (J) LIG1-depleted extracts were supplemented with wild-type xLIG1-3×Flag or xLIG1-K924W-3×Flag and analyzed by immunoblotting using the indicated antibodies. (K) The extracts from (J) were used to replicate sperm nuclei. Chromatin-bound proteins were analyzed by immunoblotting (n = 2). (L) xLIG1-depleted extracts were supplemented with wild-type xLIG1-3×Flag, xLIG1-R794L-3×Flag or xLIG1-R924W-3×Flag. Purified genomic DNA from chromatin was labeled using exonuclease-deficient Klenow fragment and α-^{32}P dCTP and separated in a denaturing agarose gel (n = 2). (M) Mock- and xLIG1-depleted extracts were analyzed by immunoblotting using the indicated antibodies. (N) The extracts from (M) were used to replicate sperm nuclei. Chromatin-bound proteins were analyzed by immunoblotting (n = 3).

Figure S2

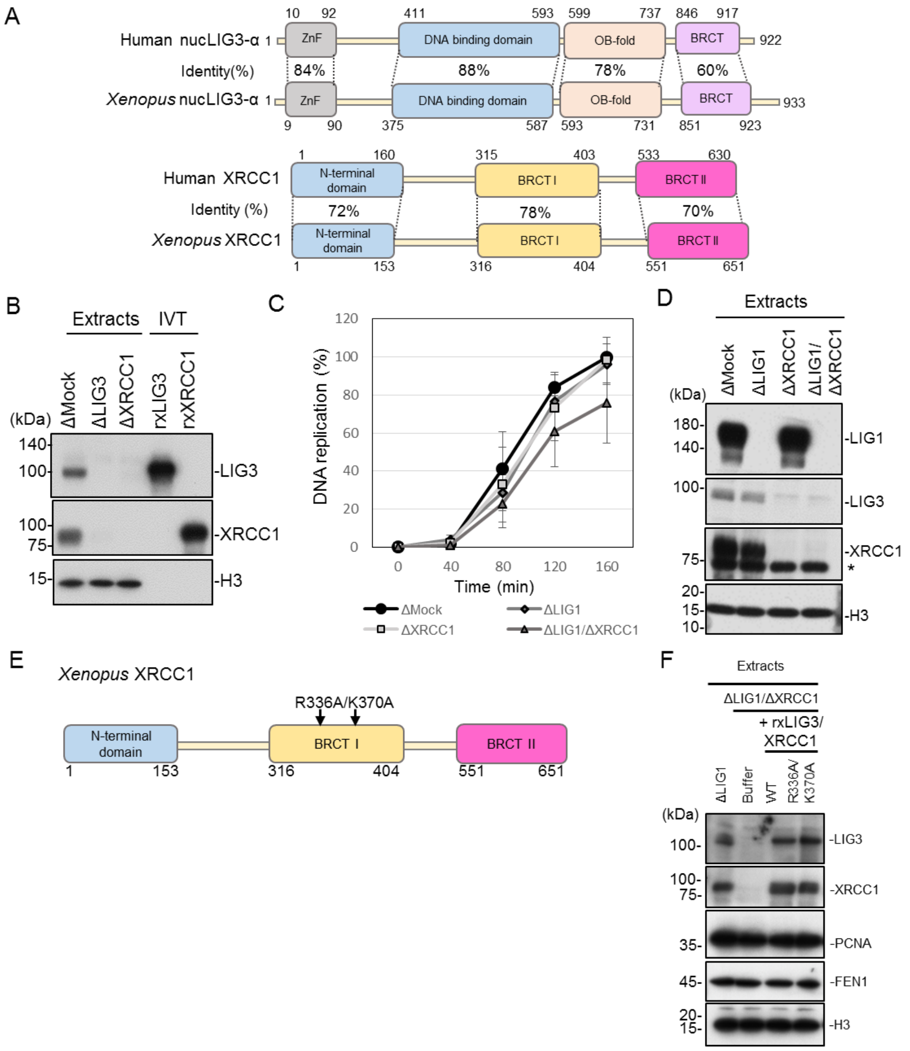

Supplementary Figure 2. (A) Domain structures of *Xenopus* and Human nuclear LIG3 and XRCC1. Nuclear LIG3 is composed of a zinc-finger (ZnF), DNA binding, oligonucleotide/oligosaccharide-binding (OB)-fold and BRCA1 C-Terminal (BRCT) domains that are well conserved in humans and *Xenopus*. XRCC1 is composed of an N-terminal domain, and BRCT I and BRCT II domains that are well conserved in humans and *Xenopus*. (B) Immunodepletion efficiency of xLIG3 and xXRCC1 from *Xenopus* egg extract. IVT; in vitro translation protein. Depleted extracts and IVT proteins were analyzed by immunoblotting using the indicated antibodies (n = 2). (C) DNA replication in xLIG1-, xXRCC1-, xLIG1/xXRCC1, and Mock-depleted extracts. The relative amounts of DNA synthesis are shown. Data shown are the average and S. D. (bars) three independent experiments. *: Significantly different by Student’s t-test at P < 0.01. (D) The extracts used in the chromatin binding experiment shown in Figure 2C were analyzed by immunoblot using the indicated antibodies. The asterisk indicates a non-specific band. (E) Sequences of the wild-type and R336A/K370A mutant BRCT I domain in xXRCC1 are shown. (F) The extracts used in the chromatin binding experiment shown in Figure 2D were analyzed by immunoblot using the indicated antibodies.

Figure S3

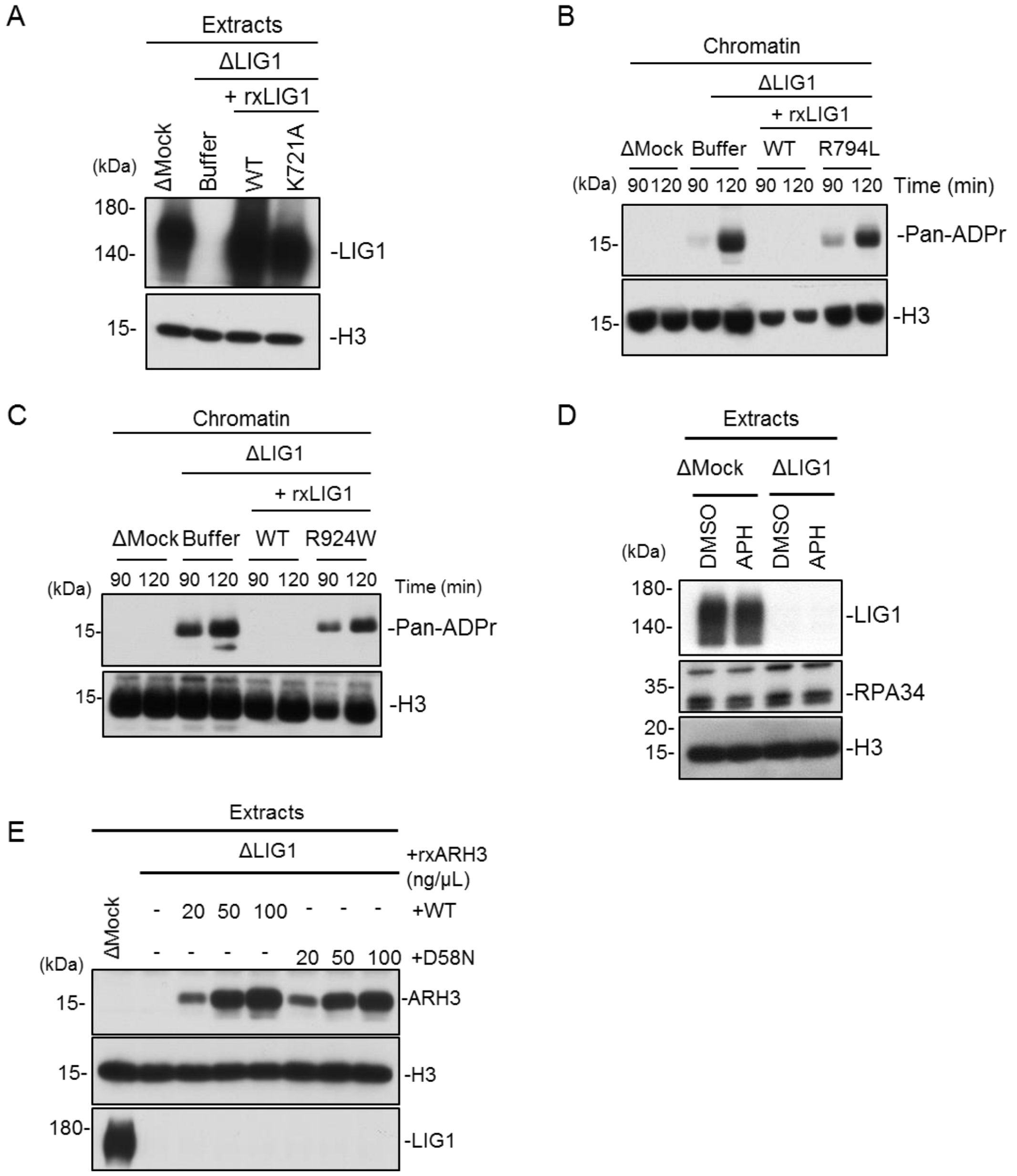

Supplementary Figure 3. (A) The extracts used in the chromatin binding experiment shown in Figure 3A were analyzed by immunoblot using the indicated antibodies. (B) xLIG1-depleted extracts were supplemented with either buffer or wild-type xLIG1-3×Flag or xLIG1-3×Flag-R794L. Chromatin-bound proteins were analyzed by immunoblotting using pan ADP-ribose detecting reagent (n = 2). (C) xLIG1-depleted extracts were supplemented with either buffer or wild-type xLIG1-3×Flag or xLIG1-3×Flag-R924W. Chromatin-bound proteins were analyzed by immunoblotting using pan ADP-ribose detecting reagent (n = 2). (D) The extracts used in the chromatin binding experiment shown in Figure 3D were analyzed by immunoblot using the indicated antibodies. (E) The extracts used in the chromatin binding experiment shown in Figure 3E were analyzed by immunoblot using the indicated antibodies.

Figure S4

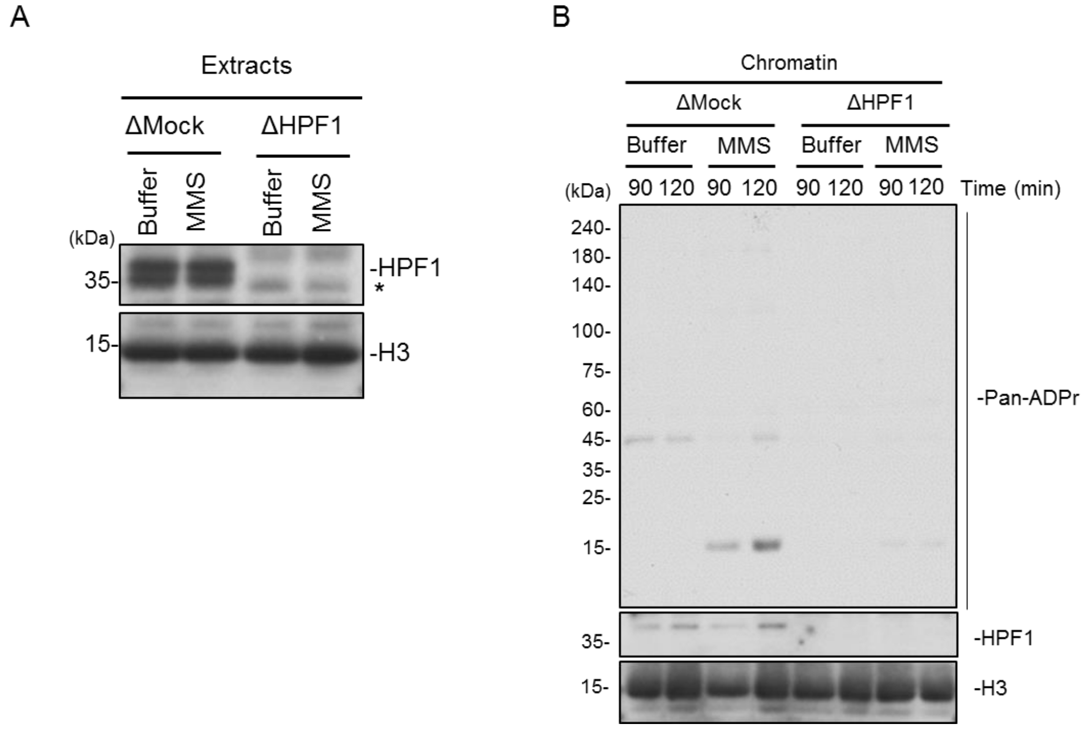

Supplementary Figure 4.

(A) Mock- and xHPF1-depleted extracts were analyzed by immunoblotting using the indicated antibodies. The asterisk indicates a non-specific band. (B) The extracts from (A) were used to replicate control or 1% MMS-treated sperm chromatin. Chromatin-bound proteins were analyzed by immunoblotting (n = 2).

Figure S5

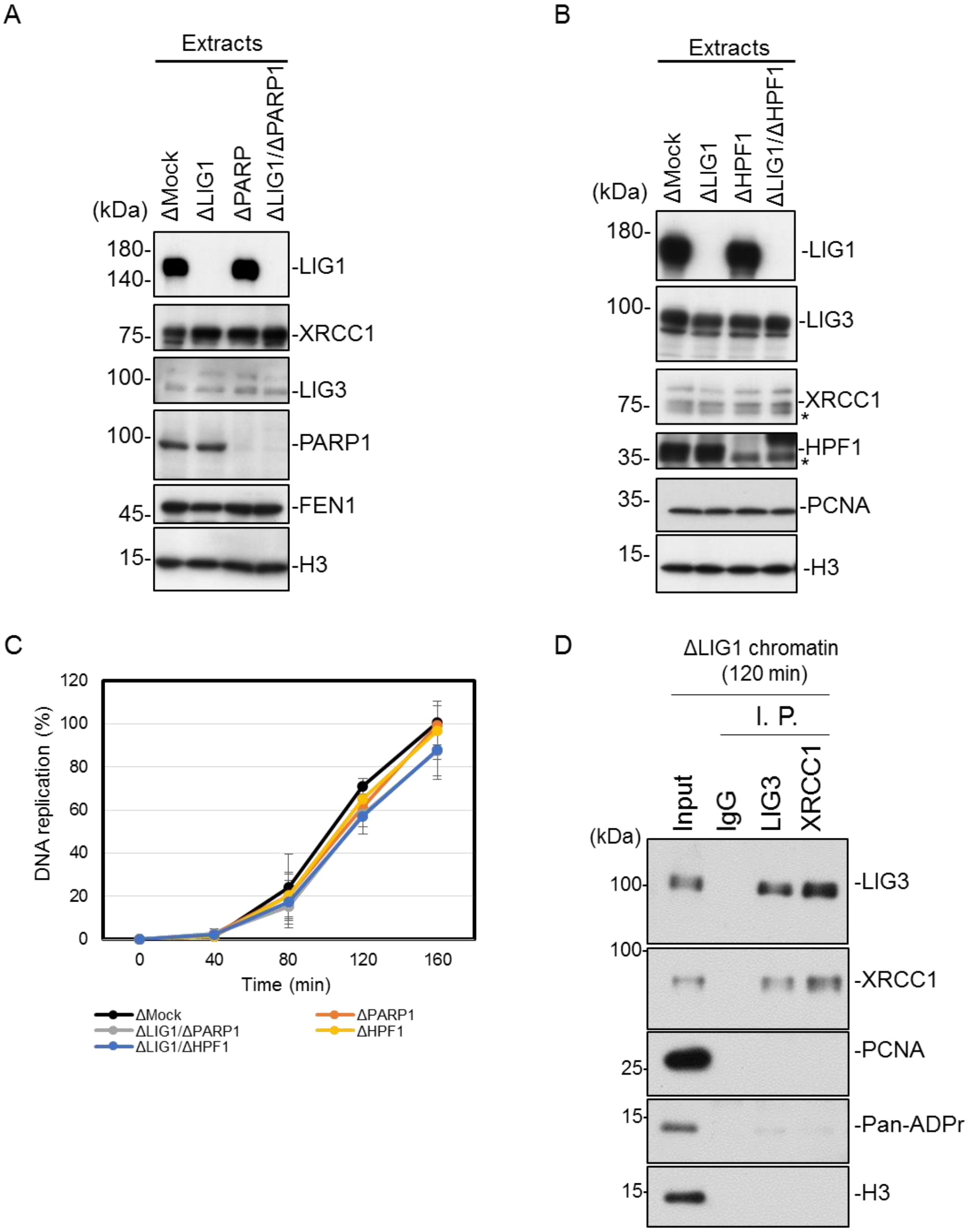

Supplementary Figure 5. (A) The extracts used in the chromatin binding experiment shown in Figure. 5A were analyzed by immunoblot using the indicated antibodies. (B) The extracts used in the chromatin binding experiment shown in Figure 5B were analyzed by immunoblot using the indicated antibodies. The asterisk indicates a non-specific band. (C) DNA replication in xPARP1-, xHPF1-, xLIG1/xPARP1, xLIG1/xHPF1, and Mock-depleted extracts. The relative amounts of DNA synthesis are shown. Data shown are the average and S. D. (bars) three independent experiments. *: Significantly different by Student’s t-test at P < 0.01. (D) Chromatin fractions from the LIG1 immunodepleted extracts was solubilized with MNase followed by immunoprecipitation with anti-xLIG3 and xXRCC1 antibodies. The resultant immunoprecipitates were analyzed (n = 2).

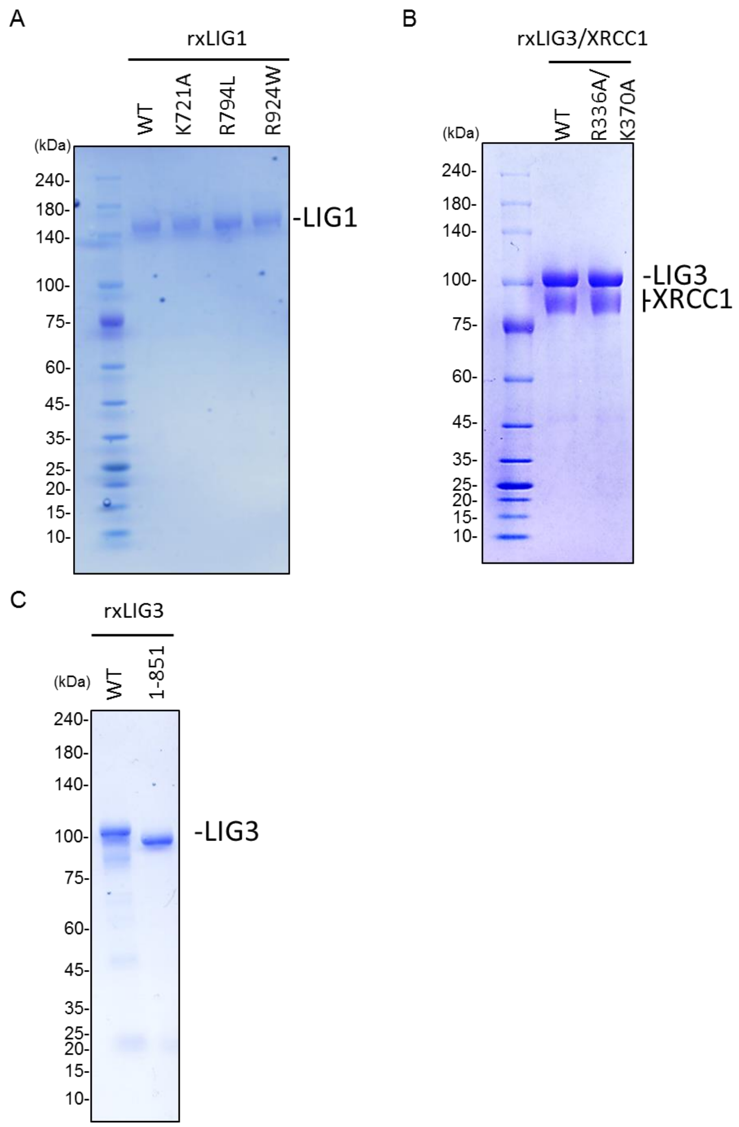

Supplementary Figure 6. (A) Wild-type LIG1, xLIG1-K721A, xLIG1-R794L, and xLIG1-R924W were expressed in Sf9 insect cells and purified using an C-terminal 3×Flag tag. The recombinant proteins were resolved by SDS－PAGE and visualized with Coomassie blue staining (n=3). (B) Wild-type 3× Flag-LIG3-xXRCC1-myc and 3×Flag-xLIG3-xXRCC1-R336AK370A-myc were co-expressed in Sf9 insect cells and purified using an N-terminal 3×Flag tag, and analyzed as in Supplementary Figure 6A (n=3). (C) Wild-type xLIG3 and xLIG3 (1-851) were expressed in Sf9 insect cells and purified using an N-terminal 3×Flag tag, and analyzed as in Supplementary Figure 6A (n=3).

Table S1

Supplementary Table 1

No.	Sequence 5'-3'	Description
1	GAAACCTTCATTTTACGGCGGGGA	xLIG1 amplification
2	ACTCCAAGGCAACACAATAGGTGG	xLIG1 amplification
3	GGCGCGGATCAGATCTCATGCAACGAACAATAAAGTC	pVL139-xLIG1-Flag3
4	GGGCCCTCTAGAATTCTACTTGTTATCGTCATCCT	pVL139-xLIG1-Flag3
5	GCATATGACGGGGAGCGTGCACAGATAC	xLig1K721A mutation
6	GTATTCACAAGTAAAGGCAGCTTCA	xLig1K721A mutation
7	GCTGCTCAGCCCAAGCTAGGGGCTGAAGTAA	xLig1F8AF9A mutation
8	GGACTTTATTGTTCGTTGCATGAGA	xLig1F8AF9A mutation
9	CTAAAGAGAAAGGATGTGGATGCATCAG	xLig1R794L mutation
10	AGTAGTCAGTACTTGAAATGGCTGA	xLig1K794L mutation
11	TGGACTGGTATCTATGGAGGCTTCTTAC	xLig1R924W mutation
12	TTTCCCTTTCCCAAGGTAAGCTCCA	xLig1R924W mutation
13	GAGTGGGCTTTCTCTGTGTTGTTG	xFEN1 amplification
14	GACATATTGTCAAAGCCTTAGCGC	xFEN1 amplification
15	TGTGTGAGTGAGGGAAATTGCAGG	xLIG3 amplification
16	GTTGATCTGTGATGCCTTCTCCTG	xLIG3 amplification
17	GGGCCCTCTAGAATTTTACACACAATTCTCATCAACAC	pVL139-xLIG3-Flag3
18	GGTGGTGTGAAGCAGAAATCTGCT	xXRCC1 amplification
19	CTTTGCACTGGCAACATCCAGTTC	xXRCC1 amplification
20	GGCGCGGATCAGATCTCATGCCTGTGATCAAACTGAAGC	pVL139-xXRCC1-Flag3
21	GGGCCCTCTAGAATTCGCCTTGGGCACCACAACGTAAGG	pVL139-xXRCC1-Flag3
22	GCAGCCGACCTCCGTGATAAAGCATTAG	xXRCC1R336A mutation
23	GAATGGATTCTGAAAGCCGCTCAGC	xXRCC1R336A mutation
24	GCATTCAGCCAGGTGAAAGCAGCGGGCG	xXRCC1R370A mutation
25	AGGGGTATTTGCAAAGGCACATATG	xXRCC1R370A mutation
26	CACGTTGTTACTGTAAGAGCCAGTGTGTTCCCTGT	xPARP1 amplification
27	CACTCCGTGATCCATCCTGCCCAACGTATG	xPARP1 amplification
28	AGCGGAAACTCCACTCTTGGTTGA	xPARG amplification
29	GCTGGACATGAGTCAGGACTTCAT	xPARG amplification
30	TTCTCAGAGAGTGCTGCTATTCGC	xHPF1 amplification
31	AAGCCATCTTCCCTCTGAAGTGAC	xHPF1 amplification
32	GCTGCGGAGCTTCCAGAGACAGATGGAAACC	xHPF1Y251AR252 mutation
33	CCCAACATCATTCTTATCCACTGGC	xHPF1Y251AR252 mutation
34	TTCTCAGAGAGTGCTGCTATTCGC	xARH3 amplification
35	AAGCCATCTTCCCTCTGAAGTGAC	xARH3 amplification
36	TGTATATGTTAAAACACTCTTTAGT	xARH3D58N mutation
37	AATGACACAGCCATGGCAAGGTCGATTG	xARH3D58N mutation
